# Towards Unbiased Fluorophore Counting in Superresolution Fluorescence Microscopy

**DOI:** 10.3390/nano13030459

**Published:** 2023-01-23

**Authors:** Oskar Laitenberger, Timo Aspelmeier, Thomas Staudt, Claudia Geisler, Axel Munk, Alexander Egner

**Affiliations:** 1 Department of Optical Nanoscopy, Institut für Nanophotonik e.V., 37077 Göttingen, Germany; 2 Institute for Mathematical Stochastics, Georg-August-University of Göttingen, 37073 Göttingen, Germany; 3 Cluster of Excellence “Multiscale Bioimaging: from Molecular Machines to Networks of Excitable Cells” (MBExC), University of Göttingen, 37075 Göttingen, Germany

**Keywords:** nanoscale characterization, fluorescence microscopy, super-resolution imaging, STORM, GSDIM, quantitative imaging

## Abstract

With the advent of fluorescence superresolution microscopy, nano-sized structures can be imaged with a previously unprecedented accuracy. Therefore, it is rapidly gaining importance as an analytical tool in the life sciences and beyond. However, the images obtained so far lack an absolute scale in terms of fluorophore numbers. Here, we use, for the first time, a detailed statistical model of the temporal imaging process which relies on a hidden Markov model operating on two timescales. This allows us to extract this information from the raw data without additional calibration measurements. We show this on the basis of added data from experiments on single Alexa 647 molecules as well as GSDIM/dSTORM measurements on DNA origami structures with a known number of labeling positions.

## 1. Introduction

Microscopy is an indispensable tool in modern biophysical, biochemical and biomedical research. The utilization of fluorescent probes [1] in particular has contributed to its development from a mainly qualitative device to an ever improving quantitative instrument. For example, fluorescence correlation spectroscopy (FCS) [2] and single-particle tracking [3] can be used to investigate the diffusion and trafficking of molecules [1]. Förster resonance energy transfer (FRET) [4], fluorescence lifetime imaging (FLIM) [5], and fluorescence recovery after photobleaching (FRAP) [6] allow the investigation of conformational changes, molecular interactions and dynamic transport phenomena [7].

Within this context, more and more researchers are interested in counting dye molecules in cells or nanostructures. In case the efficiency and stoichiometry of the labelling is known, such fluorophore numbers can be used, for example, to determine the stoichiometry of functional protein and protein-nucleic acid complexes or to improve structural models. This helps to understand functionality on the molecular scale [8,9,10,11,12,13]. Widely used methods for counting fluorescent probes are, inter alia, stepwise photobleaching and ratio comparison to fluorescent standards. Although both methods can utilize standard imaging equipment and do not require specialized analysis software, they are either limited to low molecule numbers or rely on trustworthy intensity standards [14]. In addition, these methods are limited by diffraction, which means that individual structures closer than 200–250 nm cannot be distinguished within the image.

With the advent of ’superresolution’ fluorescence microscopy (nanoscopy), the diffraction limit has been overcome by switching the signal from individual objects within a diffraction-limited region consecutively on and off [15]. Since then, these so-called optical nanoscopes enable the investigation of structures at the nanometer scale [16]. To extract quantitative information from super-resolved images it is crucial to analyze the distribution of the observed photons in time and space, which in turn depends on the photophysical properties and consequently on the underlying quantum physical mechanisms of the fluorophores used [17].

For mapping fluorophore numbers, it is important to differentiate whether switching of fluorescent signals is accomplished in a spatially controlled or in a spatially stochastic manner. If, as in STED microscopy [18], the first strategy is applied, there are usually several fluorophores within the focal spot. In this case, the frequency of simultaneous photon arrivals can be used to determine fluorophore numbers [19]. So far, however, only a very small number of fluorophores within the focal spot can be counted reliably.

Spatially stochastic switching is applied in single marker switching (SMS) nanoscopy, which subsumes many state-of-the-art techniques known as photoactivated localization microscopy (PALM) [20], stochastic optical reconstruction microscopy (STORM) [21], fluorescence photoactivation localization microscopy (FPALM) [22], PALM with independently running acquisition (PALMIRA) [23], ground state depletion microscopy followed by individual molecule return (GSDIM) [24], direct STORM (dSTORM) [25], PAINT [26] or DNA-PAINT [27]. Here, imaging is performed by assuring that with high probability only a few (random) fluorescent probes are in their on state at any time. Under excitation, these probes emit many photons, of which a fraction is detected on an array detector, e.g., an EMCCD-camera. After 1–100 ms, a fluorescent probe switches back to the off state or bleaches, so that an adjacent molecule that switches on can be read out using the same scheme. The final image is then assembled by localizing a representative number of molecules and registering them in a position histogram with an average precision which is typically in the range of several 10 nm.

Although individual fluorescent probes are localized within the SMS imaging scheme, counting fluorophores is not as straightforward as it seems to be. Fluorophores that have never been switched on during image acquisition will not be registered and therefore lead to undercounting. The simultaneous on-switching of multiple probes within a diffraction-limited region is another source of underestimation [28]. On the other hand, the photophysical properties of fluorophores can cause molecules to light up several times during image acquisition. This so-called blinking leads to multiple localizations of the same molecule, thereby counting that fluorophore several times.

A lot of effort has been put especially into resolving the overcounting problem, for example by determining the time interval $\tau_{\mathrm{crit}}$ between successive blinking events [28,29]. All localizations separated by times shorter than $\tau_{\mathrm{crit}}$ are assigned to one fluorophore. A considerable limitation of this method is the need of a priori knowledge of kinetic rates, which must be determined separately. In addition, these rates are not easily transferable from one sample to another, since the photophysical properties of the dyes used in SMS nanoscopy are usually highly dependent on the environment.

An approach to overcome this limitation deals with the stochastic nature of photophysical effects by means of a continuous time aggregated Markov model [30]. This method is not only able to determine the number of fluorophores but also the corresponding kinetic constants. The same holds true for other Markov-based approaches [31,32]. All of these approaches to counting fluorophores are based on the analysis of blinking events. Therefore, they are prone to errors if individual events are not recognized. This is particularly relevant when many fluorophores are present within a diffraction-limited region or when switching takes place on timescales that are faster or in the range of the frame acquisition rate.

Here, we develop, for the first time, a fully data-driven approach to counting fluorophores based on the analysis of time traces of fluorescence intensity. The central part of our analysis is a hidden two-timescale Markov model (HTMM) [33], which describes fluorophore switching on different timescales. It models fluorophore dynamics in fast (within one frame) and slow (between different frames) regimes, and takes into account the passage of photons through the microscope, their detection and the photo-electron amplification in the camera. This allows for the estimation of the fluorophore number based on the camera data with little need for preprocessing. In particular, the HTMM does not require a priori knowledge of the photokinetic rates, nor is it necessary to resolve or recognize individual blinking events. It is not even necessary to analyze complete time traces. Although we demonstrate its usability for the widely used Alexa Fluor 647 in a slightly modified GSDIM/dSTORM application, it is conceptually not bound to a specific dye or a group of dyes. Still, further computational challenges may have to be addressed for other fluorophore choices.

## 2. Materials and Methods

### 2.1. Fluorescence Microscope

For imaging we used a modified inverted Leica DM IRE2 microscope equipped with an oil immersion lens of a numerical aperture of 1.4 (UPLSAPO 100xO, Olympus). Two continuous wave lasers provided the excitation (LightCube Revolution, 770, R 639 nm, 1.5 W, HB-Laser) and activation (OBIS 405 LX, Coherent) light of 639 nm and 405 nm wavelength, respectively. Both laser beams were combined by a dichroic mirror (zt 442 RDC, AHF analysentechnik). A safeguard slit confined the excitation and activation light within the field of view of 33 µm × 33 µm. The intensities in the focal plane were 0.6 kW/cm${}^{2}$ for excitation and 0.07 kW/cm${}^{2}$ for activation. For detection, the fluorescence was separated from the excitation and activation light by a dichroic mirror (zt 642 rdc, AHF analysentechnik). A band pass filter (705/100 ET, AHF analysentechnik) in front of the recording EMCCD-camera (iXon 897, Andor) defined our detection bandwidth and a notch filter (zet635NF, AHF analysentechnik) blocked residual excitation light. With an electronically tunable bandpass filter (AOTFncC-VIS-TN, AA Optoelectronics) we reliably turned on the excitation laser within a time window of ~10 µs which was important for our measurement protocol. Images were acquired with the software Imspector (Max Planck Institute for Biophysical Chemistry, Göttingen, Germany).

### 2.2. Sample Preparation

DNA origami: The microscope slides (VWR) and the cover slips (VWR) were wiped with acetone (Morphisto, Offenbach am Main, Germany) and then cleaned by a plasma cleaner (femto, diener electronic) in an oxygen environment for 30 min. To immobilize the DNA origami structures (Gattaquant, Gräfelfing, Germany) we followed ’Immobilization in a flow chamber’ [34].

Cell samples: Human dermal fibroblasts were seeded on cover slips and were incubated at 37 °C and 5% carbon dioxide in cell culture medium. The cells were fixed with ice-cold methanol for 5 min and were then fluorescently labelled using standard immunolabeling protocols. For this, a primary antibody against α-tubulin (T6074, Sigma-Aldrich Chemie, Taufkirchen, Germany) and an appropriate secondary antibody conjugated to Alexa 647 (Invitrogen A21236, Thermo Fisher Scientific, Dreieich, Germany) were used. For imaging, the sample was mounted in imaging buffer on a cavity slide and sealed with a two-component silicon glue (Twinsil, Picodent, Wipperfürth, Germany).

### 2.3. Imaging Buffer

For best performance of Alexa 647 we used a similar imaging buffer as reported in [35]. It consisted of 1M Tris-HCl ph 8.0 (Life Technologies, Darmstadt, Germany), glucose oxidase 0.5 mg ml^−1^ (Sigma-Aldrich Chemie), catalase 40 µg ml^−1^ (Sigma-Aldrich Chemie), glucose 10 (*w/v*)% (Sigma-Aldrich Chemie), βME 143 mM (Sigma-Aldrich Chemie) and MgCl${}_{2}$ 12.5 mM (magnesium chloride hexahydrate, Sigma-Aldrich Chemie). The buffer was prepared just before measuring with Tris-HCL and catalase stored at 8 °C, glucose oxidase stored at −20 °C and the βME stored at 20 °C.

### 2.4. Measurement Protocol for Fluorophore Counting

We took care to protect the fluorophores on the DNA origami structures outside of our field of view (FOV) from bleaching by a safeguard slit confining the excitation and activation light within the field of view of 33 µm × 33 µm inside the observation plane. After imaging the actual FOV, we turned off all lasers and moved the specimen ~120 µm to a new FOV. Then we started a new measurement with the exposure time set to 15 ms and a dead time of 500 µs between two frames. Within the first ~25 s the camera adjusted to its background level while excitation and activation lasers were turned off. Then we started the UV laser for ~5 s to activate all fluorophores before we turned on the excitation laser. The excitation laser was timed to start ~50 µs after the EMCCD-camera began to record a new frame. The measurement was terminated after a minimum of N= 14,060 excitation-illuminated frames were recorded.

### 2.5. Data Correction and Analysis

**Figure 1:**
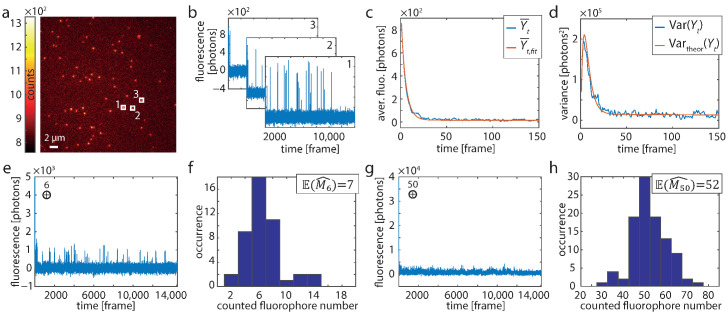
Single-molecule experiments. (**a**) First frame (t=1) of a recording of DNA origami structures exhibiting one labeling position. (**b**) Background-corrected time traces of the three 7 × 7 pixel sized evaluation regions indicated in A (white boxes). (**c**) Averaged trace and (**d**) corresponding variance from 255 origami structures (blue). The red lines in (**c**,**d**) show the fit of the HTMM and the theoretical variance, respectively. (**e**) Six traces (such as shown in b) were added at a time to generate traces of known fluorophore number *m*. (**f**) Histogram of the estimated number of dye molecules from 45 added traces modeling m=6. (**g**) Same as (**e**) for m=50. (**h**) Histogram of the estimated number of dye molecules from 100 added traces modeling m=50.

We corrected all our data for time and spatially dependent background as follows: The first frame in which the specimen was illuminated by excitation light was used to identify all pixels which detect higher signals due to origami structures, free fluorophores or other sources. In a first step, these pixels were discarded for background determination (Figure S1). A two dimensional polynomial of third degree

(1) $$\begin{array}{c} P_{t}\left(x,y\right)=a_{1}x^{2}+a_{2}y^{2}+a_{3}xy+a_{4}x+a_{5}y+a_{6}+a_{7}x^{3}+a_{8}y^{3}+a_{9}x^{2}y+a_{10}y^{2}x \end{array}$$

was fitted to not discarded pixels for every frame *t*. Hence, $P_{t}\left(x,y\right)$ is a time-dependent but spatially averaged approximation for all locations (x,y) in the FOV (Figure S1). As the counting analysis was performed locally on a 7 × 7 pixel sized evaluation region and the exact regions were discarded for determination of $P_{t}\left(x,y\right)$, we approximated the background for each evaluation region by

(2) $$\begin{array}{cc} B_{t} & =\Sigma_{i=1}^{49}P_{t}\left(x_{i},y_{i}\right)+\mu_{} \end{array}$$

where μ is an additional correction factor specific to each evaluation region. It was determined by plotting the $P_{t}\left(x,y\right)$-corrected fluorescence for each evaluation region in a histogram and fitting a Gaussian with expectation value μ and standard deviation *s* to these distributions (Figure S2). For the added traces (Figure 1E–H), a second μ was determined on the accumulated data. Finally, the corrected fluorescence for each evaluation region was calculated by

(3) $$\begin{array}{c} Y_{t}=Y_{t,\mathrm{measured}}-B_{t} \end{array}$$

The corrected values $Y_{t}$ were then evaluated using the HTMM. Note that not every frame t=1,…, 14,060 was used for evaluation, but only a subset $t_{j}$ with j=1,…,n of frames according to

(4) $$\begin{array}{c} t_{j}=\left\{\begin{array}{cc} j & \;\mathrm{for}\;1\leq j\leq 20\\ \left\lceil 19+{\left(j-19\right)}^{\beta }\right\rceil & \;\mathrm{for}\;20<j\leq n \end{array}\right. \end{array}$$

where $\beta =\frac{\log(N-19)}{\log(n-19)}$, N= 14,060 and n=4000. This was done for computational reasons: the runtime of the estimation procedure depends quadratically on the number *n* of included frames.

### 2.6. Number of Dark States Verification

We verified the number of dark states in the long-timescale model in analogy to Lee [28]: Between two blinking events, the dye is in one of its dark states. Therefore, we plotted the distribution of times between two events for 457 fluorescent traces of origami structures internally labeled with a single Alexa 647 molecule in a histogram and checked for the smallest number of exponential decays that adequately describes the histogram (Figure S3).

### 2.7. Excess Relative Variance

In order to determine the excess relative variance $\sigma_{e}^{2}$ for Alexa 647 molecules in the applied imaging buffer, we analyzed 266 background-corrected fluorescence traces of single Alexa 647 molecules which were attached to DNA origamis structures by internal labeling. For each trace, we first identified frames $t_{\mathrm{blink}}$, which belong to blinking events, by thresholding such that $Y_{t_{\mathrm{blink}}}>\mu +3s$, where {μ,s} were determined during background correction. By then rejecting the first frame of each individual blinking event, we ensured that the fluorophore was in its bright state at the beginning of all frames $t_{\mathrm{blink}}$. We calculated the excess relative variance for each trace according to $\sigma_{e}^{2}=\frac{\mathrm{Var}(Y_{t})}{E{\left(Y_{t}\right)}^{2}}-\frac{1}{E(Y_{t})}$, in which we approximated E(Y) and Var(Y) by the mean and the variance of $Y_{t_{\mathrm{blink}}}$, respectively. The results were plotted in a histogram (Figure S4) and the central position of a Gaussian fit to this histogram was then used as $\sigma_{e}^{2}$ in the calculation of the theoretical variance in Figure 1d and Figure S5.

### 2.8. Fitting Averaged Fluorescence Traces

We fitted a multi-exponential decay, Equation (8), to the average fluorescence measured over several traces for Figure 1c. The coefficients $\alpha_{k}$ and eigenvalues $\lambda_{k}$ for k=0,1,2 were obtained by a least squares fit, weighted with the inverse standard deviations of the data set. The Levenberg-Marquardt algorithm was used for minimization and the initial values were set to α = (20,000, 100, 10) and λ=(0.9,0.999,0.99999).

### 2.9. Pseudo Log-Likelihood Estimator

Our HTMM provides a stochastic process $Y_{t}$ that models the corrected time traces, depending on a set $\gamma =(m,E\left(Y\right),\sigma_{e},\alpha_{0,k},\lambda_{k},\ldots )$ of parameters that, besides the fluorophore number *m*, describe the photophysical properties of the fluorescent probe. For an observed time trace $y={\left(y_{t}\right)}_{t=1}^{n}$, however, it is impossible to calculate the full log-likelihood $l_{y}\left(\gamma \right)$ of a parameter choice γ directly, since the number of terms involved is too large. We therefore simplify $l_{y}\left(\gamma \right)$ by using a pseudo log-likelihood, which arises from a second-order approximation of $Y_{t}$ through a Gaussian process with the same expectation $\mu_{t}=E\left(Y_{t}\right)$ and temporal covariance $\Sigma_{tt^{\prime }}=E\left[\left(Y_{t}-\mu_{t}\right)\left(Y_{t^{\prime }}-\mu_{t^{\prime }}\right)\right]$. The pseudo log-likelihood is given by

(5) $$\begin{array}{c} {\tilde{l}}_{y}\left(\gamma \right)=-\frac{1}{2}\left[\left(y-\mu \right)\Sigma^{-1}\left(y-\mu \right)+\log\det\Sigma \right] \end{array}$$

and the free parameters in γ are jointly estimated by maximizing ${\tilde{l}}_{y}\left(\gamma \right)$ numerically for given traces *y*. Note that not all parameters that are needed to formulate the full HTMM are mentioned in this article, and that we employ a slightly different notation than in [33] for the parameterization. Most prominently, E(Y) is called $\theta_{1}$ and the excess variance $\sigma_{e}^{2}$ is denoted as $\theta_{3}$.

### 2.10. Simplified Estimator

In order to simplify the counting process, the pseudo log-likelihood estimator can be approximated according to Equation (10). This requires determining $Y_{1}$, the value of the time trace for t=1, and ${\bar{Y}}_{\mathrm{blink}}$, the average brightness of a fluorophore per frame, given it started the frame in its bright state. We determined $Y_{1}$ from background-corrected fluorescence traces, as depicted by the green star in Figure S6. Within the same traces, we also identified suitable blinking events, e.g., black crosses in Figure S6. For this, frames $t_{\mathrm{blink}}$ were chosen for which $Y_{t_{\mathrm{blink}}}>\mu +3s$, where {μ,s} were determined during background correction. To ensure that only frames were used, in which the fluorophore started in the bright state, the first frame of each blinking event was neglected (red dots in Figure S6). In addition, we only considered frames $t_{\mathrm{blink}}>2000$ to ensure that the blinking events originate from a single fluorophore. ${\bar{Y}}_{\mathrm{blink}}$ was then calculated as the mean of $Y_{t_{\mathrm{blink}}}$.

## 3. Results

### 3.1. Hidden Two-Timescales Markov Model

**Figure 2:**
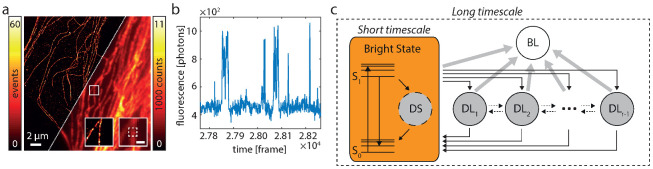
Fluorophore switching and diagram of states. (**a**) SMS (left) and widefield image (right) of Alexa 647-labeled microtubulular network. The insets show the respective images of the marked region (white box). The inset scale bar marks 500 nm. (**b**) During SMS imaging the fluorescence signal, e.g., within the white dashed box in the inset of A, fluctuates. This is due to switching of the fluorophore(s) between bright an dark states. (**c**) The general HTMM is separated into a long-timescale and a short-timescale model. Dynamics on timescales $<t_{\exp}$ are subsumed in the short-timescale model which is treated in the long-timescale model as a single bright state (orange). Excited fluorophores emit either a fluorescence photon by a transition from $S_{1}$ to $S_{0}$ or enter the short-lived dark state DS. The long-timescale model treats dynamics on timescales $\geq t_{\exp}$ and exhibits the bright, one bleached (BL) and r−1 dark states DL. Note that the bleached state can never be left and that transitions between the dark states DL are not limited to the ones depicted by the dotted arrows, but can occur between all states DL.

In SMS nanoscopy, a sample marked with a suitable dye is continuously illuminated and the emitted fluorescence is imaged on an array detector. The detector records many frames in a row at a constant exposure time $t_{\exp}$. Within any selected region, the detected fluorescence fluctuates over time as the dye molecules switch between several states during image acquisition (Figure 2a,b).

Each fluorophore can be described by a diagram of states as depicted in Figure 2c. Fluorescence is only emitted in the transitions from $S_{1}$ to $S_{0}$. All other states are “dark states” and a fluorophore which enters the bleached state will never leave it. It is important to note that the transition rates of the fluorophore span vastly different timescales, which range from ∼$10^{12}$ s${}^{-1}$ to min${}^{-1}$ or less. The exposure time of the detector, which typically lies in the range of a few 10 ms, sets a natural time grid to the experiment. Changes which occur on shorter timescales cannot be directly observed, while changes on longer timescales can. Therefore, we describe the dynamics of such a fluorophore as a time-discretized two-timescale Markov model. A short-timescale model treats fast dynamics on timescales $<t_{\exp}$ and subsumes all involved states $S_{1}$, $S_{0}$ and DS (Figure 2c). A long-timescale model treats slow dynamics on timescales $\geq t_{\exp}$. It consists of r+1 states: the bright state which reflects the net effect of running the short-timescale model for a time $t_{\exp}$, and *r* “dark states”, namely the bleached state BL and r−1 states DL. Note that states with similar lifetimes can be combined into a single state (see Remark 1 in [33]).

On the basis of this model, the background-corrected expectation value $E(Y_{t})$ and variance Var$\left(Y_{t}\right)$ of the observable fluorescence $Y_{t}$ from *m* mutually independent fluorophores within a selected region in the frames can be calculated (Theorem 4 in [33]):

(6) $$\begin{array}{cc} E(Y_{t}) & =mE\left(Y\right)\sum_{k=0}^{r-1}\left(\nu \alpha_{0,k}+\left(1-\nu \right)\alpha_{1,k}\right)\lambda_{k}^{t-1} \end{array}$$

(7) $$\begin{array}{cc} \mathrm{Var}(Y_{t}) & =\left(\left(\sigma_{e}^{2}+1\right)E\left(Y\right)+f^{2}-\frac{1}{m}E\left(Y_{t}\right)\right)E\left(Y_{t}\right), \end{array}$$

where *t* is the index of the corresponding frame, E(Y) is the expectation value of detected photons *Y* during one frame from a single fluorophore, given it started the frame in its bright state, ν is the initial fraction of dye molecules occupying the bright state at the beginning of the experiment, $\alpha_{0,k}$ and $\alpha_{1,k}$ are coefficients depending on the initial state occupation and $\lambda_{k}$ are the eigenvalues of the model’s transition matrix. Furthermore, $\sigma_{e}^{2}=\frac{\mathrm{Var}(Y)}{E{\left(Y\right)}^{2}}-\frac{1}{E(Y)}$ is the excess relative variance of *Y*, which stems from the dark state in the short-timescale model (i.e., $\sigma_{e}^{2}=0$ for no dark state DS) and $f^{2}$ is the excess noise factor of the area detector (e.g., $f^{2}=2$ for an EMCCD camera [36]).

### 3.2. Model for Alexa 647 and Single-Molecule Experiments

**Figure 3:**
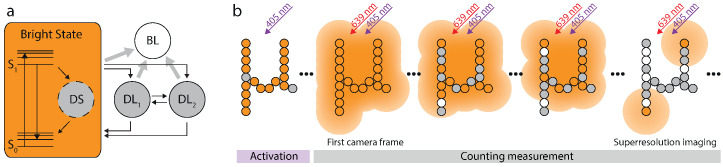
Photophysical model for Alexa 647 and imaging protocol. (**a**) The photophysical model for Alexa 647 exhibits three dark states. DS accounts for the excess variance observed in single molecule traces. DL${}_{1}$ and DL${}_{2}$ account for the triplet and an even longer living dark state respectively. (**b**) At the beginning of the measurement all fluorophores are activated by UV-illumination. Recording starts when the excitation laser is switched on. The first frames are used for counting only. Once the blinking has become sufficiently sparse, the data is also used for sub-diffraction imaging.

To verify the capability of our approach experimentally, the HTMM has to be adapted to the dye used. We decided on Alexa 647 because it is one of the most common dyes for GSDIM or dSTORM imaging. As our imaging buffer contains β-mercaptoethanol (βME, Materials and Methods), the long-timescale model for Alexa 647 exhibits two dark states (Figure 3a): the triplet state (DL${}_{1}$) and an additional dark state (DL${}_{2}$) which is presumably formed by thiol addition to the fluorophore [37]. The validity of this assumption was also verified by examining whether the distribution of residence times in one of the dark states can be described by a double exponential decay for single Alexa 647 molecules (Figure S3, Materials and Methods). The existence of the dark state (DS) in the short-timescale model is justified by an observed excess relative variance $\sigma_{e}^{2}>0$ in single-molecule time traces (Figure S4, Materials and Methods).

Alexa 647 can be switched off and on by red and UV light respectively, and is most likely in its bright state without illumination. Since it is beneficial for our analysis to know the initial state occupation ν, we first applied 405 nm on-switching light (Figure 3b). Thus, it can be safely assumed that all Alexa 647 molecules are in the bright state at the beginning of the measurement (ν=1 in Equation (6)), which is marked by the activation of the red excitation laser and the simultaneous start of the data recording. As time progresses, Alexa 647 molecules may occupy any of the indicated states and accumulate in the long-living dark and the bleached state. When enough fluorophores have entered these states such that the molecules that are in their bright state are on average much more distant from each other than the diffraction limit, their images can be discerned and thus localized. In this case, the recorded data can not only be used for counting but also for sub-diffraction imaging.

As ν=1 and r+1=4 in this Alexa 647 model, Equation (6) becomes

(8) $$\begin{array}{cc} E(Y_{t}) & =mE\left(Y\right)\sum_{k=0}^{2}\alpha_{0,k}\lambda_{k}^{t-1}=\sum_{k=0}^{2}\alpha_{k}\lambda_{k}^{t-1} \end{array}$$

with $\alpha_{k}:=mE\left(Y\right)\alpha_{0,k}$. This is a superposition of three exponential functions and has in total eight parameters. Using the constraint $\sum_{k=0}^{2}\alpha_{0,k}=1$ and applying the pseudo log-likelihood approach from [33] to numerically determine E(Y), $\alpha_{k}$ and $\lambda_{k}$ (see Materials and Methods), we can derive the number of fluorophores from Equation (8) for t=1 (first frame):

(9) $$\begin{array}{c} m=\frac{\sum_{k=0}^{2}\alpha_{k}}{E(Y)}. \end{array}$$

For the single-molecule experiments, we utilized 12-helix bundle DNA origami structures [34] which were internally labeled with a maximum of one Alexa 647-conjugated staple strand. The origami structures were immobilized on a BSA-biotin-neutravidin coated cover glass (Materials and Methods) and were spatially separated enough to be imaged individually. For imaging we started the camera ∼30 s prior to the actual data acquisition to allow its background level to stabilize. Additionally, we switched on the UV laser five seconds before the excitation laser in order to activate all fluorophores. The on-switching of the excitation was precisely synchronized to the image acquisition.

The first frame (t=1) out of such a recording is shown in Figure 1a. Background-corrected time traces were evaluated over 7 × 7 pixel sized regions centered on identified origami structures (Figure 1a,b). The temporal evolution of the fluorescence intensity is excellently described by the HTMM, as can be seen from the averaged trace of 255 molecules and the respective fit of Equation (8) for m=1 (Figure 1c and Figure S5). The theoretical variance, which is calculated from the fit result using Equation (7) and $\sigma_{e}^{2}=0.097$ (Figure S4), agrees very well with the actual variance of the traces (Figure 1d and Figure S5). As the fluorophore is certainly in the bright state at the beginning, the variance initially grows and then falls off due to the increasing probability that the dye molecule is in a dark state or bleached.

Next, we verified that a known number of dye molecules is counted correctly using the HTMM. Note that $\sigma_{e}^{2}$ was not kept fixed in this case, but was treated as a free parameter during the estimation. First, we repeatedly added m=6 traces which were randomly selected from the 255 recorded traces (Figure 1e) and evaluated them by means of the HTMM (Figure 1f). We repeated the verification also for m=50 (Figure 1g,h). The counting results show that *m* can be determined precisely, as the on average determined fluorophore numbers of $E(\hat{M_{6}})=7$ resp. $E(\hat{M_{50}})=52$ are in good agreement with the number of added traces.

### 3.3. Counting under Real Conditions

**Figure 4:**
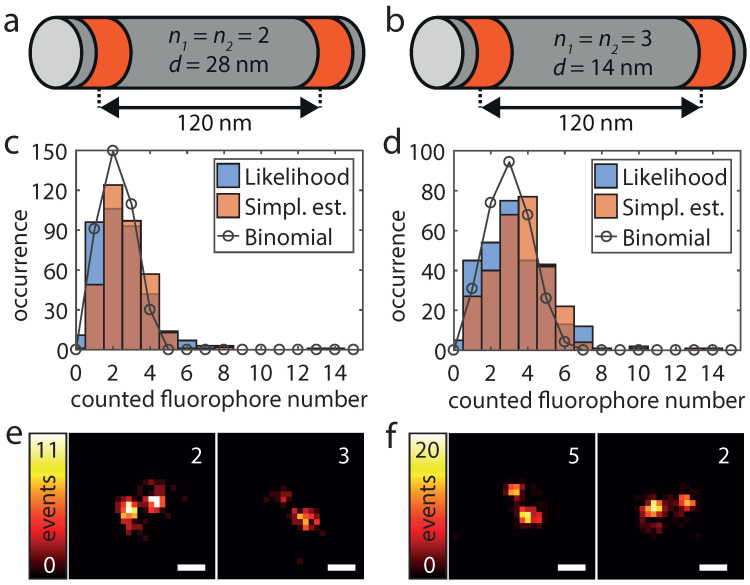
Design (**a**,**b**), dye molecule numbers (**c**,**d**) and superresolution imaging (**e**,**f**) for origami structures exhibiting 4 (**a**,**c**,**e**) and 6 (**b**,**d**,**f**) labeling positions. (**a**,**b**) The origami structures feature two marked areas (red), in which there are $n_{1}$ and $n_{2}$ labeling positions, respectively, at a nearest neighbor distance *d*. (**c**,**d**) The blue and the red histograms show the results of the pseudo log-likelihood and the simplified estimator respectively. The black circles correspond to the conditional binomial distribution with (p=0.52, *n* = 4) and (p=0.49, *n* = 6). The parameter *p* was in both cases estimated by fitting a binomial model to the histogram of the pseudo log-likelihood estimates. (**e**,**f**) For both origami designs, two super-resolved images of representative origami structures as well as the determined number of dye molecules of the pseudo log-likelihood estimator are shown. Scale bars 100 nm.

To validate the applicability of the HTMM also under real conditions, we increased the number of dye-conjugated staple strands per DNA origami structure. As depicted in Figure 4a,b, these origami structures have two marked areas which are 120 nm apart and which each features a known number of labeling positions. We name these origami designs *$n_{1}$/$n_{2}$-d*, where the first two numbers indicate the number of labeling positions per marked area and the last number indicates the nearest neighbor distance between labeling positions. The first design (2/2-28) exhibits therefore four labeling positions and within each area the two labeling positions exhibit a mutual distance of 28 nm (Figure 4a). The second design (3/3-14) has an additional labeling position in the center of each area which changed the minimum mutual distance of labeling positions to 14 nm and the overall number of labeling positions to six (Figure 4b). Since the traces are recorded directly and disturbing influences such as background signal or detector noise do not cumulate, we expect the counting to work even better than with the added single molecule traces. As the labeling yield *p* is not 100%, the number of fluorophores per origami structure (*k*) should be binomially distributed according to $B(k|p,n=n_{1}+n_{2})$. Because unlabeled origami structures cannot be detected and were therefore not analyzed, the counted number of molecules then follows K∼B(·|p,n) conditioned on {K>0}. As shown in Figure 4c,d, the determined number of fluorophores of 380 (2/2-28) resp. 297 (3/3-14) analyzed origami structures corresponds very well to a conditional binomial distribution with n=4, p=0.52 resp. n=6, p=0.49. In addition, the counted number of dye molecules can easily be mapped to the super-resolved image. It should be noted that the number of localizations per individual fluorophore can vary greatly due to the statistic nature of the blinking events (Figure 4e,f).

Interestingly, using our recording protocol, the number of dye molecules can be estimated relatively easily. For this, we substitute the summation over the coefficients $\alpha_{k}$ in Equation (9) by the value of the time trace for t=1 and E(Y) by the average intensity ${\bar{Y}}_{\mathrm{blink}}$ within the single molecule blinks:

(10) $$\begin{array}{c} m\approx \frac{Y_{1}}{{\bar{Y}}_{\mathrm{blink}}} \end{array}$$

According to the definition of E(Y) the fluorophore must be in its bright state at the beginning of the frame which is generally not the case for the first frame of a blinking event. We therefore neglected the first frame of such an event when determining ${\bar{Y}}_{\mathrm{blink}}$ (Materials and Methods). The number of dye molecules according to this simplified estimator is also shown in Figure 4.

## 4. Discussion

In this study, we followed an entirely new approach to determine fluorophore numbers in PALM/ STORM microscopy. In contrast to previous studies which evaluate blinking events, we used a complete statistical model of the imaging process. Our model describes the recorded time traces directly, which makes our approach insensitive to a number of errors, such as the non-recognition or incorrect allocation of blinking events.

The principle idea behind the HTMM is to divide the state diagram of the fluorophore in two parts: a long-time model captures the changes of long-living fluorophore states from frame to frame, while a short-time model, which incorporates states with quick transitions, determines the photon statistics for one frame. For estimation of the fluorophore number and other model parameters, we use a pseudo-likelihood based approach, where the HTMM is approximated by a Gaussian stochastic process [33].

The HTMM is extremely general and can easily be adapted to other dyes. Although the diagram of states used is photophysically motivated, it does not have to strictly represent the precise photophysics of the fluorophore. Rather, it represents a class of molecules, since, for example, states with similar lifetimes can be combined into a single one. In addition, dye molecules with the same number of states but with different transition rates can be described by the same model, since all necessary parameters are solely estimated from the experimental data.

Bringing the fluorophores into a well-defined initial state by means of an optimized recording protocol can significantly simplify data evaluation, as fewer parameters have to be determined. In addition, data analysis can be further simplified by only analyzing a representative selection of time points. Here, for example, we only used 4000 out of the 14,060 recorded frames for analysis, which reduced the required processing time accordingly. Furthermore, the fluorophore number can also be assessed by using a simplified estimator, which only requires knowledge of the intensity at the beginning of the experiment as well as the average brightness of a blinking event.

Finally, we would like to note that the HTMM can also be easily applied to other forms of fluorescence microscopy, such as widefield or confocal microscopy, as it does not depend on the analysis of single fluorophore blinks.

## Data Availability

The data that support the findings of this study are available from the authors on reasonable request.

## Supplementary Materials

The following supporting information can be downloaded at: https://www.mdpi.com/article/10.3390/nano13030459/s1, Figure S1: First step of background correction; Figure S2: Second step of background correction; Figure S3: Histogram of the time spent in one of the dark states for single Alexa Fluor 647 molecules attached to DNA origami structures; Figure S4: Histogram of excess relative variances calculated from background-corrected fluorescence traces of single Alexa 647 molecules attached to a DNA origami structure; Figure S5: Average and variance of 255 background-corrected time traces of single Alexa 647 molecules attached to a DNA origami structure; Figure S6: Determination of $Y_{1}$ and ${\bar{Y}}_{\mathrm{blink}}$ for the simplified estimator from a fluorescence trace $Y_{t}$.
