# Supplementary material for: Towards Unbiased Fluorophore Counting in Superresolution Fluorescence Microscopy
Source: Nanomaterials (Basel). 2023 Jan 23;13(3):459. doi: 10.3390/nano13030459 (PMC9921631; doi:10.3390/nano13030459)
Supplement: Supplementary file 1 [file nanomaterials-13-00459-s001.zip › nanomaterials-2032776-supplemetary updated2.pdf]

Supplementary Materials

Towards Unbiased FluorophoreCounting in Superresolution Fluorescence Microscopy

Oskar Laitenberger[1,†], Timo Aspelmeier[2,†], Thomas Staudt[2], Claudia Geisler[1], Axel Munk[2] and Alexander Egner[1,*]

1 Department of Optical Nanoscopy, Institut für Nanophotonik e.V., Gottingen, Germany; oskar.laitenberger@ifnano.de (O.L.); claudia.geisler@ifnano.de (C.G.)
2 Institute for Mathematical Stochastics, Georg-August-University of Göttingen, Gottingen, Germany; timo.aspelmeier@mathematik.uni-goettingen.de (T.A.); thomas.staudt@uni-goettingen.de (T.S.); munk@math.uni-goettingen.de (A.M.)
* Correspondence: alexander.egner@ifnano.de
† These authors contributed equally to this work.

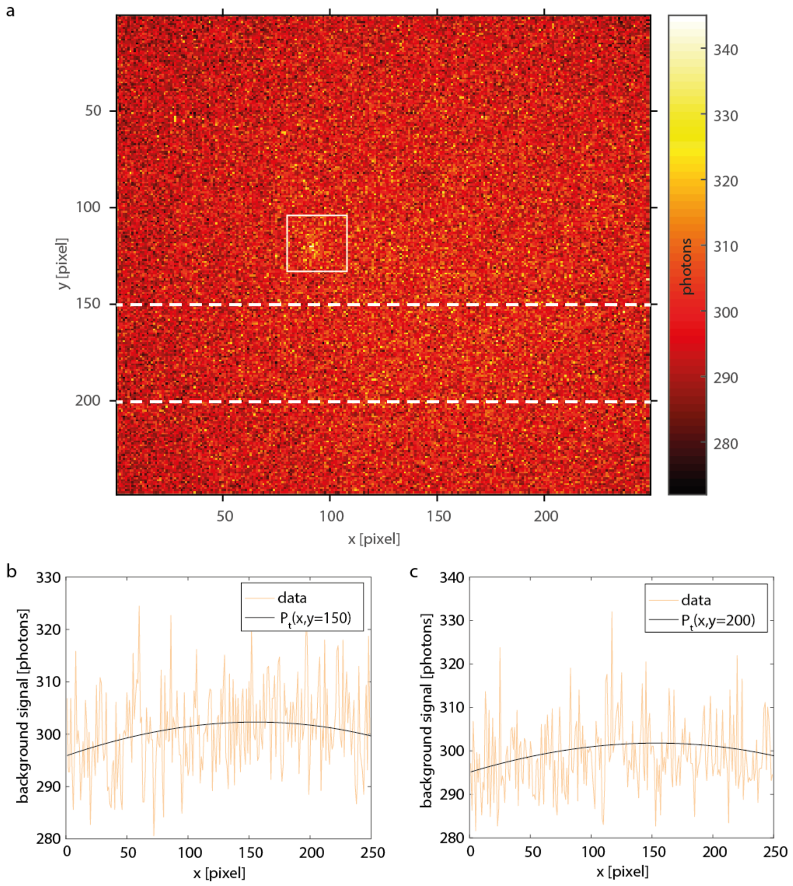

Figure S1. First step of background correction. (a) Single frame without any fluorescence signal. The spatial dependency of the background can be sensed. The elevated signal within the white box stems from an under-suppressed reflection from the activation laser. All data within the box have been discarded during the first step of background determination. **(b, c)** Fit of the two dimensional polynomial of third degree, $P_t(x, y)$. The fit is shown for different slices through the field of view $(y = 150, 200)$.

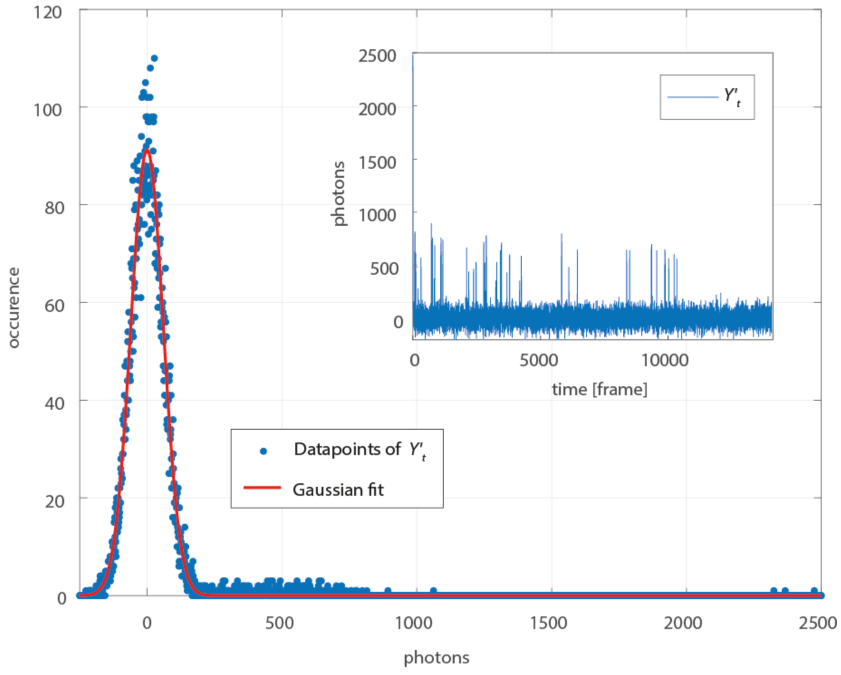

Figure S2. Second step of background correction. The histogram shows the frequency of occurrence of different photon numbers within the $P_t(x, y)$-corrected fluorescence trace (Y'_t) for one evaluation region. The additional correction factor μ and its standard deviation s are determined by fitting a Gaussian (red) to the noise mode. The inlaid picture shows Y'_t.

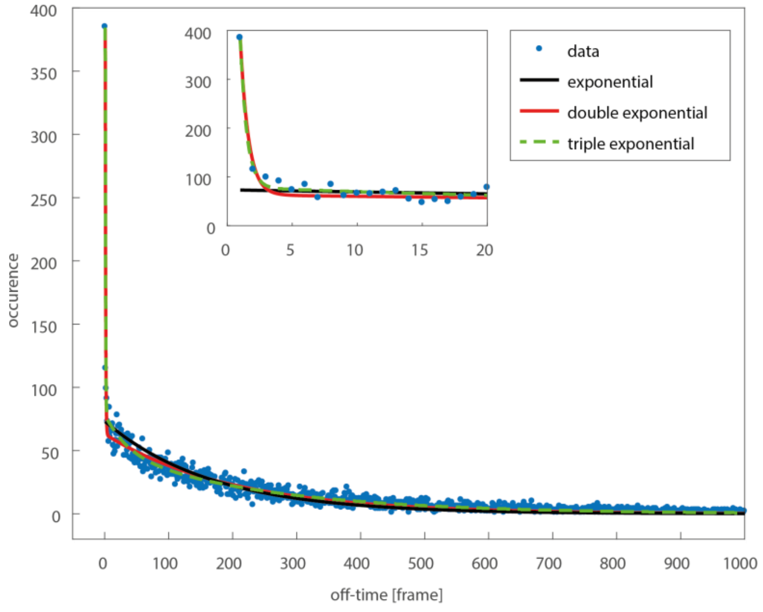

Figure S3. Histogram of the time spent in one of the dark states for single Alexa Fluor 647 molecules attached to DNA origami structures. Fits of single (black), double (red) and triple (green, dashed) exponential decays to the data are also shown. The double exponential decay describes the observed times adequately. The triple exponential decay gives very similar results.

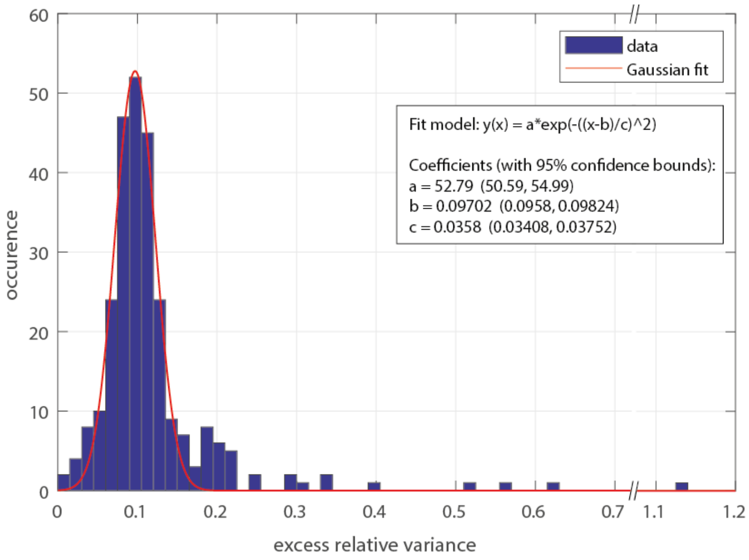

Figure S4. Histogram of excess relative variances calculated from background-corrected fluorescence traces of single Alexa 647 molecules attached to a DNA origami structure. The Gaussian fit (red) to the histogram is centered at $x \approx 0.097$.

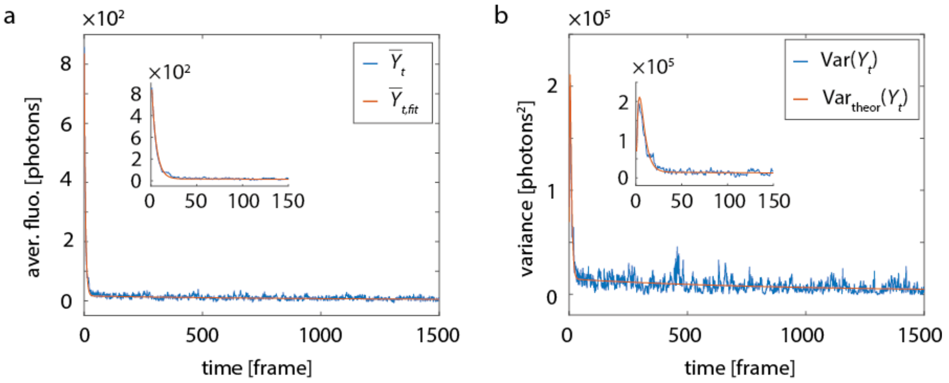

Figure S5. Average (a) and variance (b) of 255 background-corrected time traces of single Alexa 647 molecules attached to a DNA origami structure (blue). The red lines in (a) and (b) show the fit of the HTMM and the theoretical variance, respectively. The data are the same as in Figure 1c,d in the main text, but are shown here over a 10x larger time range in order to better visualize the quality of the fit on longer time scales. The inset shows the same data range as in Figure c,d in the main text.

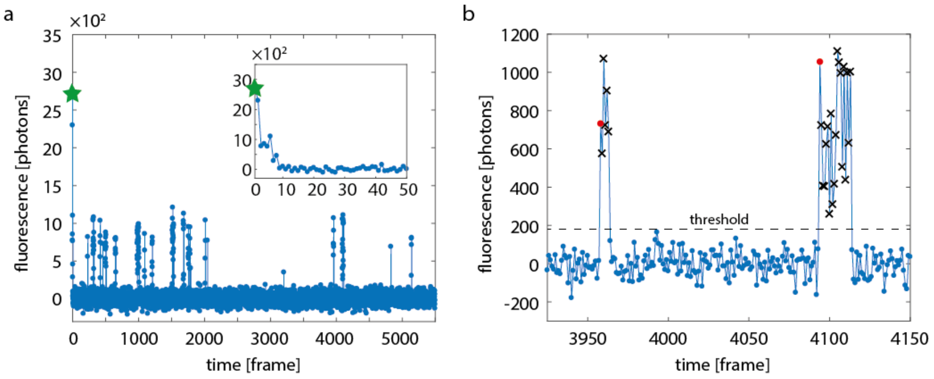

Figure S6. Determination of Y_1 and $\bar{Y}_{\text{blink}}$ for the simplified estimator from a fluorescence trace Y_t. (a) Y_1 is given by the first value of Y_t (green star). (b) $\bar{Y}_{\text{blink}}$ can be determined by calculating the mean of all values above the threshold (black cross). Due to the definition of $\mathbb{E}(Y)$ the first data point of each burst (red) has to be discarded. To ensure that the bursts originate from single molecule events, only frames with $t > 2000$ were considered.
